# Bibliometric Analysis of Neurology Articles Published in General Medicine Journals

**DOI:** 10.1001/jamanetworkopen.2021.5840

**Published:** 2021-04-15

**Authors:** Mitch Wilson, Margaret Sampson, Nick Barrowman, Asif Doja

**Affiliations:** 1 Department of Neurology, Beth Israel Deaconess Medical Center, Boston, Massachusetts; 2 Children’s Hospital of Eastern Ontario, Ottawa, Ontario, Canada; 3 Children’s Hospital of Eastern Ontario Research Institute, Ottawa, Ontario, Canada

## Abstract

**Importance:**

A significant portion of neurology literature is published in general medicine journals. Despite this, a detailed examination of publication patterns of neurology articles in these journals has not yet been carried out.

**Objective:**

To examine the publication patterns of neurology articles in general medicine journals during a 10-year period using a bibliometric approach.

**Design, Setting, and Participants:**

This cross-sectional bibliometric analysis identified the top 5 general medicine journals using the 2017 Journal Citations Report. Four other medical subspecialties (ie, immunology, endocrinology, gastroenterology, and pulmonology) were selected for comparison of publication patterns with neurology. Using MEDLINE, the 5 journals were searched for articles published between 2009 and 2018 that were indexed with the following MeSH terms: *nervous system diseases, immune system diseases, endocrine system diseases, gastrointestinal diseases,* and *respiratory tract diseases. *Data analysis was conducted from February 2019 to December 2020.

**Main Outcomes and Measures:**

Publications were characterized by journal, specialty, and study design. These variables were used for comparison of publication numbers.

**Results:**

The general medicine journals with the 5 highest journal impact factors (JIF) were *New England Journal of Medicine *(*NEJM*; JIF 79.3), *Lancet* (JIF 53.3), *JAMA* (JIF 47.7), *BMJ* (JIF 23.6), and *PLOS Medicine* (JIF 11.7). Our bibliometric search yielded 3719 publications, of which 1098 (29.5%) were in neurology. Of these 1098 neurology publications, 317 (28.9%) were published in *NEJM*, 205 (18.7%) in *Lancet*, 284 (25.9%) in *JAMA*, 214 (19.5%) in *BMJ*, and 78 (7.1%) in *PLOS Medicine*. Randomized clinical trials were the most frequent neurology study type in general medicine journals (519 [47.3%]). The number of publications in each of the other specialties were as follows: immunology, 817; endocrinology, 633; gastroenterology, 353; and pulmonology, 818.

**Conclusions and Relevance:**

The results of this study provide some guidance to authors regarding where they may wish to consider submitting their neurology research. Compared with other specialties, neurology-based articles are published more frequently in general medicine journals.

## Key Points

**Question:**

What are the publication patterns for neurology publications in general medicine journals, and how do they compare with other specialties?

**Findings:**

In this cross-sectional bibliometric analysis of the top 5 most cited general medicine journals, the *New England Journal of Medicine* (*NEJM*) published more neurology articles than other journals. In the top 5 general medicine journals, there were more publications in neurology than in immunology, endocrinology, gastroenterology, or pulmonology.

**Meaning:**

In this study, neurology articles were published most often in *NEJM*, and general medicine journals published more articles in neurology than in other medical specialties.

## Teaser

This cross-sectional study examines the publication patterns of neurology articles in general medicine journals during a 10-year period using a bibliometric approach.

## Introduction

There has been considerable growth in scientific publications over time,^1^ including in the field of neurology, and researchers are faced with a number of options regarding where to publish their articles. Neurology-focused research articles (NFRAs) are published in a number of journal types, including general neurology journals, neurology subspecialty journals, and general medicine journals (GMJs). However, there has been little examination of the precise publication patterns of NFRAs, specifically how frequently GMJs publish neurology articles and how the publication of neurology articles compares with other specialties. Bibliometric analysis can provide data regarding productivity rates, publication patterns, and publication characteristics by providing statistical descriptions of publications based on, in part, the premise that the published literature of a field embodies the field’s knowledge.^2,3^ It uses computerized analytic techniques with the individual publication as the unit of analysis, drawing data from sources such as MEDLINE, Google Scholar, or Journal Citation Reports (JCR; Clarivate Analytics).^4^ Bibliometric methods have been used to explore the productivity of researchers, institutions, and countries within given subject areas; to examine research trends and emphases in various disciplines; and to guide policy decision making.^5,6,7,8^ The JAMA Network has served as a medium for publishing bibliometric research in various medical specialties, including dermatology, ophthalmology, critical care, and obstetrics and gynecology (OBGYN),^9,10,11,12^ but bibliometric research related to neurology has not been extensively published. Therefore, our goal was to examine publication patterns of NFRAs in GMJs using a bibliometric approach and to compare these publication patterns with other medical subspecialties.

## Methods

The institutional review board of the Children’s Hospital of Eastern Ontario deemed that ethical approval was not necessary, as this was a pure bibliometric study. This study followed the Strengthening the Reporting of Observational Studies in Epidemiology (STROBE) reporting guideline.^15^

### Journal Selection

The top 5 GMJs were identified using the JCR. The JCR generates a list of journals ranked by journal impact factor (JIF). The JCR data was filtered by the following: (1) selected categories, Medicine, General, and Internal; (2) selected JCR year, 2017; (3) selected editions, Science Citation Index Expanded (SCIE); and (4) selected category scheme, Web of Science. We then identified the 5 journals with the highest JIFs that met the following criteria: (1) GMJ, (2) accepting adult and pediatric articles, and (3) publishing primary research reports (ie, not exclusively review articles). We then obtained each journal’s 2017 JIF,^13^ reflecting the mean number of citations to articles published in that journal.

### Specialty Selection

We sought to compare the publication patterns seen in neurology with 4 other medical subspecialties. To establish these 4 additional disciplines, we examined the JCR categories and selected the 4 specialties with the highest number of total citations in the JCR 2017 report that also met the following criteria: (1) the specialty must be relevant to all age groups, excluding, eg, gerontology and pediatrics, and (2) the specialty must be clinically focused. When an article was indexed with MeSH terms for more than 1 specialty, we included the article in each of those specialties.

### Bibliometric Search Strategy

On April 24, 2019, using Ovid MEDLINE, we searched the 5 journals for articles published between 2009 and 2018 that were indexed with the following MeSH terms: *nervous system diseases, immune system diseases, endocrine system diseases, gastrointestinal diseases,* and *respiratory tract diseases*. We further restricted the search to records with abstracts and excluded commentaries, editorials, historical articles, and letters. Records were downloaded into Reference Manager version 11 (Thomas Reuters).

### Study Designs and Review of Classification Accuracy

Our search included MEDLINE classification for the following study designs: randomized clinical trials (RCTs), clinical trials, systematic reviews, case reports, cohort studies, cross-sectional studies, case-control studies, and comparative studies. A hierarchical filtering process was used to ensure all study types were mutually exclusive and that each article was only indexed for 1 study type. We reviewed a random 5% sample of the records to ensure classification accuracy for specialty and study type.

### Statistical Analysis

Data analysis was conducted from February 2019 to December 2020. Models for the number of publications by year, journal, and specialty were constructed. Poisson models were initially considered; however, due to the presence of overdispersion, these models were replaced by quasi-Poisson models. The starting point was a model including main effects for year, journal, and specialty as well as all 2-way interactions. When the evidence for an interaction was weak (*P* > .10), that interaction was removed, and the reduced model was fitted. Based on the final model, estimated marginal means by year, journal, and specialty were computed. Within journals, contrasts between the 4 other specialties (immunology, endocrinology, gastroenterology, and pulmonology) and neurology were estimated, averaging over years, and are displayed as ratios with 95% CIs, adjusted for multiple testing using the multivariate *t*-distribution method.^14^ All tests were 2-tailed, and analyses were conducted in R version 4.0.4 (R Project for Statistical Computing).

## Results

The JCR 2017 report revealed that the 5 GMJs with the highest JIFs were *New England Journal of Medicine *(*NEJM*; JCR rank 1; JIF 79.3), *Lancet* (JCR rank 2; JIF 53.3), *JAMA* (JCR rank 3; JIF 47.7), *BMJ* (JCR rank 4; JIF 23.6), and *PLOS Medicine* (JCR rank 9; JIF 11.7) (Table 1). The overall range was 11.7 to 79.3, with a mean of 43.1 (95% CI, 10.3-76.0) and a median (interquartile range) of 47.7 (23.6-53.3). In comparison, 5 of top neurology journals by JIF (*Lancet Neurology, JAMA Neurology, Brain, Annals of Neurology,* and *Neurology*) had a range of 3.1 to 27.1, with a mean of 8.7 (95% CI, 0.0-21.5) and a median (interquartile range) of 4.0 (3.7-5.5). The 4 selected specialties used for comparison with neurology were immunology, endocrinology, gastroenterology, and pulmonology.

**Table 1.** Top 5 General Medicine Journals in the Specialty of Neurology, Including Distribution by Journal and by Study Design

Characteristic	No.					Total, No. (%)
	*NEJM*	*Lancet*	*JAMA*	*BMJ*	*PLOS Medicine*	
JCR rank	1	2	3	4	9	NA
Journal impact factor	79.3	53.3	47.7	23.6	11.7	NA
Study design						
RCT	227	133	105	45	56	519 (47)
Clinical trial	10	7	1	1	2	21 (2)
Systematic review	0	12	26	37	9	84 (8)
Case reports	35	6	20	0	1	62 (6)
Cohort study	31	34	114	103	49	331 (30)
Cross-sectional study	1	1	9	8	4	23 (2)
Case-control study	7	8	6	12	4	37 (3)
Comparative study	6	4	3	8	0	21 (2)
Total, No. (%)	317 (29)	205 (19)	284 (26)	214 (19)	78 (7)	1098 (100)

Abbreviations: JCR, Journal Citations Report; *NEJM*, *New England Journal of Medicine*; NA, not applicable; RCT, randomized clinical trial.

### Review of Classification Accuracy

A total of 186 of the 3719 (5.0%) results were reviewed. Accuracy was 96.2% (95% CI, 93.5%-98.9%) by specialty and 96.8% (95% CI, 94.3%-99.3%) by study design.

### Bibliometric Search Results

Our bibliometric search yielded 3719 publications, of which 1098 (29.5%) were in neurology. Of these 1098 neurology publications, 317 (28.9%) were published in *NEJM*, 205 (18.7%) in *Lancet*, 284 (25.9%) in *JAMA*, 214 (19.5%) in *BMJ*, and 78 (7.1%) in *PLOS Medicine* (mean, 220; 95% CI, 105-334). RCTs were the most frequent neurology study type in general medicine journals, accounting for 519 of 1098 publications (47.3%). Table 1 shows the distribution of NFRAs in the GMJs as well as study types. *NEJM *published 0 meta-analyses, while *BMJ *published 37; *BMJ *published 0 case studies, while *NEJM* published 35. Table 2 compares the total number of NFRA with other specialties and subdivides these by study type.

**Table 2.** Distribution of Publications in Each of the 5 Specialties by Journal and Study Design

Characteristic	No.					Total, No. (%)
	Neurology	Immunology	Endocrinology	Gastroenterology	Pulmonology	
Journal						
* NEJM*	317	289	159	91	242	1098 (30)
* Lancet*	205	187	102	76	145	715 (19)
* JAMA*	284	128	153	109	221	895 (24)
* BMJ*	214	80	154	62	144	654 (18)
* PLOS Medicine*	78	133	65	15	66	357 (10)
Total, No. (%)	1098 (30)	817 (22)	633 (17)	353 (9)	818 (22)	3719 (100)
Study design						
RCT	519	460	276	176	426	1857 (50)
Clinical trial	21	21	4	6	12	64 (2)
Systematic review	84	48	67	25	71	295 (8)
Case report	62	47	29	21	39	198 (5)
Cohort study	331	194	206	102	209	1042 (28)
Cross-sectional study	23	18	21	5	23	90 (2)
Case-control study	37	14	17	13	18	99 (3)
Comparative study	21	15	13	5	20	74 (2)

Abbreviations: *NEJM*, *New England Journal of Medicine*; RCT, randomized clinical trial.

### Comparison of the Yearly Number of Publications Between Neurology and Other Specialties

The number of publications in each of the other specialties were as follows: immunology, 817; endocrinology, 633; gastroenterology, 353; and pulmonology, 818. A quasi-Poisson model was fitted for number of publications per year, including main effects for year, journal, and subspecialty and all 2-way interactions. There was evidence of an interaction between year and journal (χ^2^_36_ = 63.2; *P* = .003) and between journal and subspecialty (χ^2^_52_ = 121.5; *P* < .001). However, there was little evidence of an interaction between year and subspecialty (χ^2^_36_ = 26.9; *P* = .86), and this interaction was removed from the model. Using this reduced model, the ratio of number of publications per year for each subspecialty compared with neurology was estimated within each journal (Figure). NFRAs were published overall more in GMJs than other subspecialities. However, some exceptions were noted for individual GMJs; for example, *PLOS Medicine* published more immunology articles than NFRA.

**Figure.**
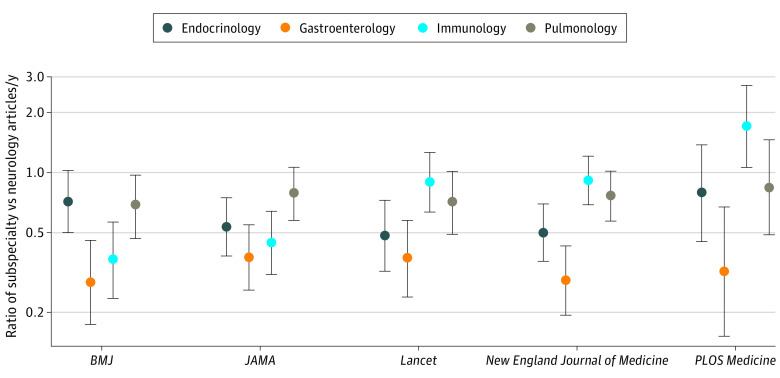
Estimated Ratio of Number of Publications per Year for Each Subspecialty vs Neurology Within Each Journal Dots represent the estimated ratio of mean number of publications for each subspecialty versus neurology. Error bars represent 95% CIs. There were 250 combinations of year, journal, subspecialty.

## Discussion

Our results indicate that *NEJM* published the most neurology studies compared with other GMJs. GMJs tend to publish neurology articles more than other specialties; the exception to this was *PLOS Medicine*, which published immunology most frequently of the specialties we studies.

It is apparent that the top GMJs have higher JIFs than neurology-focused journals. In our study, the range, mean, and median JIFs for the top 5 GMJs were 11.7 to 79.3, 43.1, and 47.7, respectively. In 5 of the top neurology journals by JIF (*Lancet Neurology, JAMA Neurology, Brain, Annals of Neurology,* and *Neurology*), the range, mean, and median JIF were 3.1 to 27.1, 8.7, and 4.0, respectively. This trend is not limited to the specialty of neurology. For example, a bibliometric study examining the publication patterns of OBGYN articles found that the top-cited articles published in nonspecialty journals were more frequently cited than those in OBGYN journals.^12^ One reason that is likely driving higher JIFs in GMJs vs neurology-focused journals, and is a reason for authors to preferentially publish in these journals, is that the neurology articles published in GMJs tend to be applicable to clinicians and researchers other than neurologists.

It was noteworthy that *NEJM* did not publish any systematic reviews. This is consistent with a prior analysis of the prevalence of review articles in GMJs,^16^ which demonstrated that *NEJM* did publish narrative reviews but did not publish any systematic reviews. Unlike the other 4 journals examined in our study that did publish systematic reviews, *NEJM* does not endorse the Preferred Reporting Items for Systematic Reviews and Meta-analyses (PRISMA) reporting guideline.^17,18^

There was a decrease in the number of publications of neurology (and other subspecialties) with decreasing JIF. This likely is likely because GMJs journals such as *NEJM*, *JAMA*, and *Lancet* all have higher JIFs than specialty journals, including neurology-specific journals. However, with decreasing impact factors, authors essentially have more choice. While they could submit to *PLOS Medicine*, with a JIF of 11.7, they could also choose to submit to a neurology-specific journal with a similar or even higher impact factor (eg, *JAMA Neurology* has an impact factor of 12.3).^19^

There are many factors for neurology researchers to consider when deciding where to publish their work. Researchers typically want their research to reach both a large and interested audience, which can be accomplished by publishing in a journal with a high JIF and whose scope is relevant to the topic of the publication, respectively. Whether the JIF or the scope of the journal is more important is likely highly variable among individual researchers and the nature of the publication. Further qualitative research may help to elucidate the most important factors used by authors to determine where to publish their neurology-based research.

### Limitations

Our study has limitations. One limitation of our study, inherent to any bibliometric approach, was that we did not examine individual article records, except those in the random sample we examined to verify the accuracy of indexing. Rather, we relied on indexing in MEDLINE for classifications. Nonetheless, accuracy of the 5% sample we manually verified was very high, and thus, we can be confident that the results achieved through bibliometric methods are a true reflection of the GMJ literature. It also would have been interesting to see which neurological subspecialties were most heavily represented in high-impact GMJs, although this analysis is beyond the scope of the present study.

Using the JIF to select the 5 most cited GMJs has both advantages and limitations. JIF is widely used because it is an established metric that allows for comparison of journal impact within disciplines and is designed to reduce bias toward larger journals that publish more articles annually. The main limitation of the JIF is that it does not reflect the number of citations (or impact) of individual articles but rather the number of citations of the journal. In this sense, highly cited articles published in journals with low JIF are deemed to have a lower impact in the literature than articles with fewer citations published in journals with higher JIF. Similarly, individual articles can make a disproportionate contribution to the overall JIF; as such, the JIF may not provide the most accurate assessment of the quality of the individual articles that are published within a journal. Additionally, JIF does not consider journals citing their own publications (ie, self-citation) and the context of the citation (ie, positive vs negative citation).

## Conclusions

In this study, neurology articles were published most often in *NEJM*, and more neurology articles were published in GMJs than other medical specialties. These results may provide some guidance to authors regarding where they may wish to consider submitting their neurology research, depending on the type of study design used.
